# 6-Nitro-2,3-bis­(thio­phen-2-yl)quinoxaline

**DOI:** 10.1107/S2414314620002035

**Published:** 2020-02-14

**Authors:** Jorge F. de Freitas, Shayne Brown, James S. Oberndorfer, Guy Crundwell

**Affiliations:** a Department of Chemistry & Biochemistry, Central Connecticut State University, 1619 Stanley Street, New Britain, CT 06053, USA; Institute of Biotechnology CAS, Czech Republic

**Keywords:** crystal structure, quinoxaline, thio­phene

## Abstract

The title compound, C_16_H_9_N_3_O_2_S_2_, was synthesized *via* a condensation reaction in refluxing acetic acid. One thienyl ring is nearly coplanar with the quinoxaline unit [dihedral angle = 3.29 (9)°], the other makes an angle of 83.96 (4)°.

## Abstract 2

The structure of the title nitro­bis­(thio­phen-2-yl)quinoxaline has been determined at 298 K.

## Graphical abstract

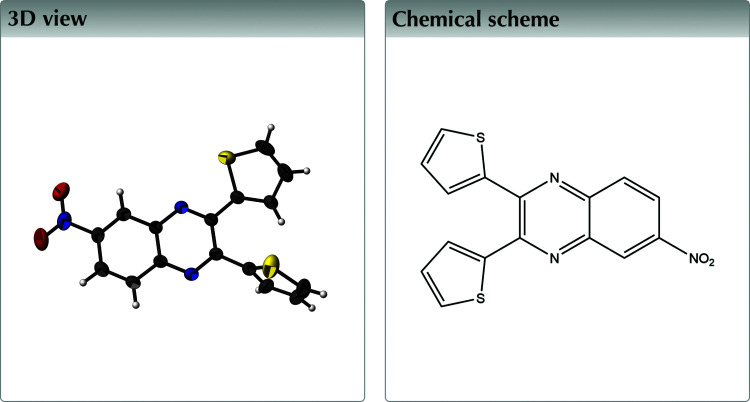

## Structure description

**Figure 1:**
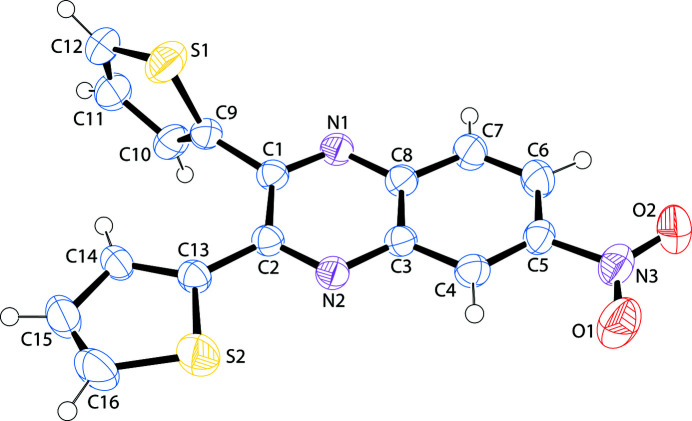
A view of 6-nitro-2,3-bis­(thio­phen-2-yl)quinoxaline (Farrugia, 2012 ▸). Displacement ellipsoids are drawn at the 50% probability level.

**Figure 2:**
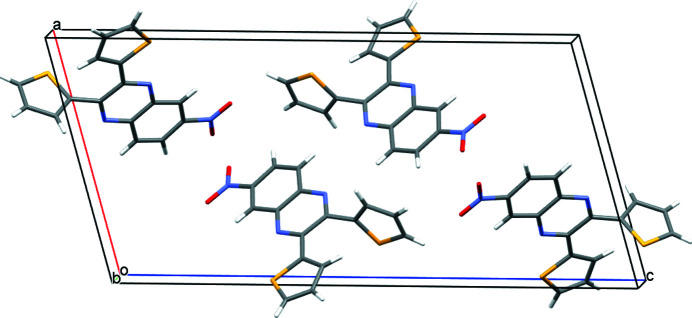
A view of the unit cell of 6-nitro-2,3-bis­(thio­phen-2-yl)quinoxaline along (010).

6-Nitro-2,3-bis­(thio­phen-2-yl)quinoxaline crystallizes in space group *P*2_1_/c. All bond lengths and angles are within expected values. Unlike in the related mol­ecule 5-nitro-2,3-bis­(thio­phen-2-yl)quinoxaline (de Freitas *et al.*, 2020 ▸), one thienyl ring and the nitro group in the title compound are nearly coplanar with the quinoxaline moiety. The nitro group makes a dihedral angle of 7.76 (14)° with respect to the mean plane of the quinoxaline unit. A survey of the literature on other 6-nitro­quinoxalines reveals that the nitro group is routinely nearly coplanar. The two thienyl rings make dihedral angles of 83.96 (4) and 3.29 (9)°, for the rings with S1 and S2 respectively, with the mean plane of the quinoxaline unit. The coplanar thienyl ring sulfur atom is closer in proximity to the quinoxaline nitro­gen atom, in the *trans* arrangement of Du & Zhao (2003 ▸). The other thienyl ring is nearly perpendicular to the plane of the quinoxaline; barely adopting the aforementioned authors *cis* arrangement. There are no inter­molecular inter­actions of consequence. An *ORTEP* view is shown in Fig. 1 ▸ and a view of the unit cell along (010) is shown in Fig. 2 ▸.

## Synthesis and crystallization

2-Thio­phene­carboxaldehyde was condensed to 2,2′-thenoin (Crundwell *et al.*, 2002 ▸) followed by oxidation to 2,2′-thenil (Crundwell *et al.*, 2003 ▸). The nitro­phenyl­enedi­amines were used as purchased from Sigma–Aldrich.

In a 100 ml round-bottom flask, 2.22 g of 2,2′-thenil (10.0 mmol) and 1.52 g of 4-nitro-1,2-phenyl­enedi­amine were added to 50 ml of concentrated acetic acid. The solution was refluxed with stirring for 18 h. The solution was cooled to room temperature and neutralized with 6 *M* NaOH. The solution was again cooled then filtered. The resulting solid was filtered and washed with cold water then dried. The yield of the yellow product was 3.10 g (92%), m.p. 474 K. ^1^H NMR (CDCl_3_, 300 MHz): δ = 7.10 (*m*, 2H), 7.43 (*m*, 2H), 7.61 (*m*, 2H), 8.20 (*d*, 1H), 8.49 (*dd*, 1H), 8.98 (*d*, 1H); ^13^C NMR (CDCl_3_, 300 MHz): δ = 123.4, 125.2, 127.8, 127.9, 130.2, 130.3, 130.7, 139.3, 140.5, 140.8, 143.0, 147.8, 148.7, 149.3.

## Refinement

Crystal data, data collection and structure refinement details are summarized in Table 1 ▸.

## Supplementary Material

Crystal structure: contains datablock(s) I, global. DOI: 10.1107/S2414314620002035/ff4034sup1.cif

Structure factors: contains datablock(s) I. DOI: 10.1107/S2414314620002035/ff4034Isup2.hkl

Supporting information file. DOI: 10.1107/S2414314620002035/ff4034Isup3.cml

CCDC reference: 1983315

Additional supporting information:  crystallographic information; 3D view; checkCIF report

## Figures and Tables

**Table 1:** Experimental details

Crystal data	
Chemical formula	C_16_H_9_N_3_O_2_S_2_
*M*_r_	339.38
Crystal system, space group	Monoclinic, *P*2_1_/*c*
Temperature (K)	293
*a*, *b*, *c* (Å)	11.7649 (4), 5.3386 (2), 24.3536 (8)
β (°)	105.610 (3)
*V* (Å^3^)	1473.18 (9)
*Z*	4
Radiation type	Mo *K*α
μ (mm^−1^)	0.37
Crystal size (mm)	0.40 × 0.30 × 0.18

Data collection	
Diffractometer	Oxford Diffraction Xcalibur, Sapphire3
Absorption correction	Multi-scan (*CrysAlis PRO*; Oxford Diffraction, 2009 ▸)
*T*_min_, *T*_max_	0.871, 1.000
No. of measured, independent and observed [*I* > 2σ(*I*)] reflections	12501, 5931, 3620
*R*_int_	0.020
(sin θ/λ)_max_ (Å^−1^)	0.802

Refinement	
*R*[*F* ^2^ > 2σ(*F* ^2^)], *wR*(*F* ^2^), *S*	0.062, 0.199, 1.06
No. of reflections	5931
No. of parameters	208
H-atom treatment	H-atom parameters constrained
Δρ_max_, Δρ_min_ (e Å^−3^)	0.51, −0.33

Computer programs: *CrysAlis CCD* and *CrysAlis RED* (Oxford Diffraction, 2009 ▸), *SHELXS2014* (Sheldrick, 2008 ▸), *SHELXL2014* (Sheldrick, 2008 ▸), *ORTEP-3 for Windows* (Farrugia, 2012 ▸) and *OLEX2* (Bourhis *et al.*, 2015 ▸).

## full crystallographic data

### Crystal data

**Table:**

C_16_H_9_N_3_O_2_S_2_	*D*_x_ = 1.530 Mg m^−^^3^
*M**_r_* = 339.38	Melting point: 474 K
Monoclinic, *P*2_1_/*c*	Mo *K*α radiation, λ = 0.71073 Å
*a* = 11.7649 (4) Å	Cell parameters from 4982 reflections
*b* = 5.3386 (2) Å	θ = 4.2–34.7°
*c* = 24.3536 (8) Å	µ = 0.37 mm^−^^1^
β = 105.610 (3)°	*T* = 293 K
*V* = 1473.18 (9) Å^3^	Block, yellow
*Z* = 4	0.40 × 0.30 × 0.18 mm
*F*(000) = 696	

### Data collection

**Table:**

Oxford Diffraction Xcalibur, Sapphire3 diffractometer	5931 independent reflections
Radiation source: fine-focus sealed tube	3620 reflections with *I* > 2σ(*I*)
Graphite monochromator	*R*_int_ = 0.020
phi and ω scans	θ_max_ = 34.7°, θ_min_ = 4.2°
Absorption correction: multi-scan (CrysAlisPro; Oxford Diffraction, 2009)	*h* = −18→18
*T*_min_ = 0.871, *T*_max_ = 1.000	*k* = −8→7
12501 measured reflections	*l* = −36→38

### Refinement

**Table:**

Refinement on *F*^2^	Primary atom site location: structure-invariant direct methods
Least-squares matrix: full	Secondary atom site location: difference Fourier map
*R*[*F*^2^ > 2σ(*F*^2^)] = 0.062	Hydrogen site location: inferred from neighbouring sites
*wR*(*F*^2^) = 0.199	H-atom parameters constrained
*S* = 1.06	*w* = 1/[σ^2^(*F*_o_^2^) + (0.0958*P*)^2^ + 0.397*P*] where *P* = (*F*_o_^2^ + 2*F*_c_^2^)/3
5931 reflections	(Δ/σ)_max_ = 0.001
208 parameters	Δρ_max_ = 0.51 e Å^−^^3^
0 restraints	Δρ_min_ = −0.33 e Å^−^^3^

### Special details

**Table:**

Geometry. All esds (except the esd in the dihedral angle between two l.s. planes) are estimated using the full covariance matrix. The cell esds are taken into account individually in the estimation of esds in distances, angles and torsion angles; correlations between esds in cell parameters are only used when they are defined by crystal symmetry. An approximate (isotropic) treatment of cell esds is used for estimating esds involving l.s. planes.
Refinement. Refinement of F^2^ against ALL reflections. The weighted R-factor wR and goodness of fit S are based on F^2^, conventional R-factors R are based on F, with F set to zero for negative F^2^. The threshold expression of F^2^ > 2sigma(F^2^) is used only for calculating R-factors(gt) etc. and is not relevant to the choice of reflections for refinement. R-factors based on F^2^ are statistically about twice as large as those based on F, and R- factors based on ALL data will be even larger. H atoms were included in calculated positions with C-H distances of 0.93 Å and were included in the refinement in riding motion approximation with U_iso_ = 1.2 of the carrier atom.

### Fractional atomic coordinates and isotropic or equivalent isotropic displacement parameters (Å^2^)

**Table:**

	*x*	*y*	*z*	*U*_iso_*/*U*_eq_	
S1	0.16791 (7)	0.82603 (13)	0.52719 (3)	0.0622 (2)	
S2	0.02258 (6)	1.15002 (14)	0.31003 (3)	0.0592 (2)	
O1	0.30694 (18)	0.1759 (4)	0.20797 (8)	0.0669 (5)	
O2	0.43910 (18)	−0.0612 (4)	0.26181 (9)	0.0663 (5)	
N2	0.20929 (14)	0.7957 (3)	0.33911 (7)	0.0357 (3)	
N1	0.36211 (14)	0.6752 (3)	0.44590 (7)	0.0369 (3)	
N3	0.37406 (17)	0.1200 (4)	0.25404 (8)	0.0463 (4)	
C1	0.27918 (15)	0.8444 (4)	0.44089 (8)	0.0328 (4)	
C2	0.20193 (15)	0.9143 (3)	0.38578 (7)	0.0323 (3)	
C3	0.29042 (16)	0.6090 (3)	0.34485 (8)	0.0322 (3)	
C4	0.29411 (17)	0.4671 (4)	0.29649 (8)	0.0373 (4)	
H4	0.2428	0.5008	0.2609	0.045*	
C5	0.37543 (17)	0.2785 (4)	0.30344 (8)	0.0361 (4)	
C6	0.45704 (19)	0.2266 (4)	0.35510 (9)	0.0445 (5)	
H6	0.5124	0.0998	0.3576	0.053*	
C7	0.45498 (19)	0.3639 (4)	0.40225 (9)	0.0442 (5)	
H7	0.5094	0.3318	0.4370	0.053*	
C8	0.37005 (16)	0.5542 (4)	0.39796 (8)	0.0343 (4)	
C9	0.26743 (17)	0.9512 (4)	0.49523 (8)	0.0369 (4)	
C10	0.3322 (2)	1.1441 (5)	0.52755 (8)	0.0452 (5)	
H10	0.3919	1.2336	0.5179	0.054*	
C11	0.2935 (2)	1.1845 (5)	0.57763 (9)	0.0529 (6)	
H11	0.3249	1.3084	0.6042	0.063*	
C12	0.2088 (2)	1.0296 (5)	0.58287 (10)	0.0567 (6)	
H12	0.1756	1.0320	0.6135	0.068*	
C13	0.11360 (16)	1.1131 (4)	0.37766 (8)	0.0351 (4)	
C14	0.08780 (17)	1.2923 (4)	0.41567 (9)	0.0380 (4)	
H14	0.1266	1.3051	0.4542	0.046*	
C15	−0.0063 (2)	1.4494 (5)	0.38591 (12)	0.0545 (6)	
H15	−0.0363	1.5785	0.4035	0.065*	
C16	−0.0479 (2)	1.3957 (5)	0.33041 (13)	0.0607 (7)	
H16	−0.1089	1.4836	0.3057	0.073*	

### Atomic displacement parameters (Å^2^)

**Table:**

	*U*^11^	*U*^22^	*U*^33^	*U*^12^	*U*^13^	*U*^23^
S1	0.0796 (5)	0.0583 (4)	0.0622 (4)	−0.0135 (3)	0.0425 (3)	−0.0078 (3)
S2	0.0561 (4)	0.0676 (4)	0.0461 (3)	0.0207 (3)	0.0002 (2)	0.0051 (3)
O1	0.0714 (12)	0.0817 (14)	0.0453 (10)	0.0018 (10)	0.0120 (8)	−0.0174 (9)
O2	0.0820 (13)	0.0516 (10)	0.0741 (12)	0.0145 (10)	0.0364 (10)	−0.0087 (9)
N2	0.0348 (7)	0.0401 (8)	0.0312 (7)	0.0049 (6)	0.0069 (6)	0.0012 (6)
N1	0.0352 (7)	0.0417 (9)	0.0318 (8)	0.0048 (7)	0.0057 (6)	0.0023 (6)
N3	0.0508 (10)	0.0464 (10)	0.0482 (10)	−0.0082 (8)	0.0248 (8)	−0.0092 (8)
C1	0.0307 (8)	0.0359 (9)	0.0317 (8)	0.0016 (7)	0.0080 (6)	0.0030 (7)
C2	0.0301 (8)	0.0337 (8)	0.0330 (8)	0.0012 (7)	0.0083 (6)	0.0026 (7)
C3	0.0313 (8)	0.0343 (8)	0.0313 (8)	0.0004 (7)	0.0091 (6)	0.0021 (7)
C4	0.0376 (9)	0.0420 (10)	0.0314 (8)	0.0030 (8)	0.0075 (7)	0.0020 (7)
C5	0.0374 (9)	0.0363 (9)	0.0381 (9)	−0.0019 (7)	0.0164 (7)	−0.0034 (7)
C6	0.0410 (10)	0.0443 (11)	0.0481 (11)	0.0107 (9)	0.0120 (8)	0.0010 (9)
C7	0.0389 (9)	0.0520 (12)	0.0389 (10)	0.0155 (9)	0.0056 (7)	0.0039 (9)
C8	0.0304 (8)	0.0392 (9)	0.0325 (8)	0.0026 (7)	0.0070 (6)	0.0023 (7)
C9	0.0382 (9)	0.0409 (10)	0.0318 (9)	0.0066 (8)	0.0097 (7)	0.0045 (7)
C10	0.0478 (11)	0.0561 (13)	0.0310 (9)	−0.0003 (10)	0.0091 (8)	−0.0035 (8)
C11	0.0652 (14)	0.0565 (14)	0.0345 (11)	0.0066 (12)	0.0091 (9)	−0.0050 (9)
C12	0.0774 (17)	0.0574 (14)	0.0433 (12)	0.0127 (13)	0.0302 (11)	0.0004 (10)
C13	0.0317 (8)	0.0354 (9)	0.0374 (9)	0.0026 (7)	0.0082 (7)	0.0032 (7)
C14	0.0367 (9)	0.0332 (9)	0.0433 (10)	0.0069 (7)	0.0092 (7)	0.0039 (7)
C15	0.0541 (13)	0.0411 (12)	0.0739 (17)	0.0140 (10)	0.0271 (12)	0.0075 (11)
C16	0.0462 (12)	0.0614 (15)	0.0714 (17)	0.0211 (11)	0.0103 (11)	0.0219 (13)

### Geometric parameters (Å, º)

**Table:**

S1—C9	1.707 (2)	C5—C6	1.390 (3)
S1—C12	1.703 (3)	C6—H6	0.9300
S2—C13	1.7171 (19)	C6—C7	1.368 (3)
S2—C16	1.696 (3)	C7—H7	0.9300
O1—N3	1.223 (3)	C7—C8	1.409 (3)
O2—N3	1.216 (3)	C9—C10	1.392 (3)
N2—C2	1.324 (2)	C10—H10	0.9300
N2—C3	1.361 (2)	C10—C11	1.428 (3)
N1—C1	1.311 (2)	C11—H11	0.9300
N1—C8	1.359 (2)	C11—C12	1.327 (4)
N3—C5	1.467 (3)	C12—H12	0.9300
C1—C2	1.453 (2)	C13—C14	1.420 (3)
C1—C9	1.481 (3)	C14—H14	0.9300
C2—C13	1.461 (3)	C14—C15	1.422 (3)
C3—C4	1.411 (3)	C15—H15	0.9300
C3—C8	1.408 (2)	C15—C16	1.339 (4)
C4—H4	0.9300	C16—H16	0.9300
C4—C5	1.368 (3)		
			
C12—S1—C9	91.82 (12)	N1—C8—C3	120.51 (17)
C16—S2—C13	92.06 (12)	N1—C8—C7	119.36 (16)
C2—N2—C3	117.91 (15)	C3—C8—C7	120.06 (17)
C1—N1—C8	117.97 (16)	C1—C9—S1	119.81 (15)
O1—N3—C5	118.3 (2)	C10—C9—S1	111.71 (16)
O2—N3—O1	124.1 (2)	C10—C9—C1	128.42 (19)
O2—N3—C5	117.62 (19)	C9—C10—H10	125.0
N1—C1—C2	121.85 (17)	C9—C10—C11	110.0 (2)
N1—C1—C9	115.35 (16)	C11—C10—H10	125.0
C2—C1—C9	122.76 (16)	C10—C11—H11	122.9
N2—C2—C1	120.08 (16)	C12—C11—C10	114.1 (2)
N2—C2—C13	116.08 (16)	C12—C11—H11	122.9
C1—C2—C13	123.84 (16)	S1—C12—H12	123.8
N2—C3—C4	119.05 (16)	C11—C12—S1	112.30 (18)
N2—C3—C8	121.41 (17)	C11—C12—H12	123.8
C8—C3—C4	119.54 (17)	C2—C13—S2	116.76 (14)
C3—C4—H4	121.0	C14—C13—S2	111.13 (14)
C5—C4—C3	118.00 (17)	C14—C13—C2	132.09 (17)
C5—C4—H4	121.0	C13—C14—H14	125.1
C4—C5—N3	118.04 (18)	C13—C14—C15	109.80 (19)
C4—C5—C6	123.28 (18)	C15—C14—H14	125.1
C6—C5—N3	118.66 (19)	C14—C15—H15	122.8
C5—C6—H6	120.4	C16—C15—C14	114.3 (2)
C7—C6—C5	119.27 (19)	C16—C15—H15	122.8
C7—C6—H6	120.4	S2—C16—H16	123.7
C6—C7—H7	120.1	C15—C16—S2	112.70 (18)
C6—C7—C8	119.78 (19)	C15—C16—H16	123.7
C8—C7—H7	120.1		
